# Polymeric Nanoparticles of *Pistacia lentiscus* var. *chia* Essential Oil for Cutaneous Applications

**DOI:** 10.3390/pharmaceutics12040353

**Published:** 2020-04-14

**Authors:** Ilianna Vrouvaki, Eleni Koutra, Michael Kornaros, Konstantinos Avgoustakis, Fotini N. Lamari, Sophia Hatziantoniou

**Affiliations:** 1 Laboratory of Pharmaceutical Technology, Department of Pharmacy, University of Patras, 26504 Patras, Greece; iliannavrouvaki@gmail.com (I.V.); avgoust@upatras.gr (K.A.); 2 Laboratory of Biochemical Engineering and Environmental Technology (LBEET), Department of Chemical Engineering, University of Patras, 26504 Patras, Greece; ekoutra@chemeng.upatras.gr (E.K.); kornaros@chemeng.upatras.gr (M.K.); 3 INVALOR: Research Infrastructure for Waste Valorization and Sustainable Management, University Campus, 26504 Patras, Greece; 4 Laboratory of Pharmacognosy and Chemistry of Natural Products, Department of Pharmacy, University of Patras, 26504 Patras, Greece; flam@upatras.gr

**Keywords:** nanotechnology, *Pistacia lentiscus* var. *chia* essential oil, mastic gum, nanoparticles, poly(lactic acid), antimicrobial activity, topical application

## Abstract

Polymeric nanoparticles (NPs) encapsulating *Pistacia lentiscus* L. var. *chia* essential oil (EO) were prepared by a solvent evaporation method, in order to obtain a novel carrier for administration on the skin. The specific EO exhibits antimicrobial and anti-inflammatory properties thus stimulating considerable interest as a novel agent for the treatment of minor skin inflammations. The incorporation into nanoparticles could overcome the administration limitations that inserts the nature of the EO. Nanoparticles were prepared, utilizing poly(lactic acid) (PLA) as shell material, due to its biocompatibility and biodegradability, while the influence of surfactant type on NPs properties was examined. Two surfactants were selected, namely poly(vinyl alcohol) (PVA) and lecithin (LEC) and NPs’ physicochemical characteristics i.e. size, polydispersity index (PdI) and ζ-potential were determined, not indicating significant differences (*p* > 0.05) between PLA/PVA-NPs (239.9 nm, 0.081, -29.1 mV) and PLA/LEC-NPs (286.1 nm, 0.167, −34.5 mV). However, encapsulation efficiency (%EE) measured by GC-MS, was clearly higher for PLA/PVA-NPs than PLA/LEC-NPs (37.45% vs. 9.15%, respectively). Moreover PLA/PVA-NPs remained stable over a period of 60 days. The in vitro release study indicated gradual release of the EO from PLA/PVA-NPs and more immediate from PLA/LEC-NPs. The above findings, in addition to the SEM images of the particles propose a potential structure of nanocapsules for PLA/PVA-NPs, where shell material is mainly consisted of PLA, enclosing the EO in the core. However, this does not seem to be the case for PLA/LEC-NPs, as the results indicated low EO content, rapid release and a considerable percentage of humidity detected by SEM. Furthermore, the Minimum Inhibitory Concentration (MIC) of the EO was determined against *Escherichia coli* and *Bacillus subtilis*, while NPs, however did not exhibit considerable activity in the concentration range applied. In conclusion, the surfactant selection may modify the release of EO incorporated in NPs for topical application allowing its action without interfering to the physiological skin microbiota.

## 1. Introduction

Dating back to the first century AD, ancient Greek physicians (Hippocrates, Dioscorides, Galenos) reported the therapeutic properties of *Pistacia lentiscus* L. var. *chia* (mastic tree of Chios), an evergreen shrub growing on the Greek island of Chios. The resin of the plant (mastic gum), is well known for its medicinal uses, especially for the treatment of stomach disorders, preservation of oral hygiene and wound healing action [1,2,3]. Since 2015 the Committee on Herbal Medicinal Products (HMPC) of European Medicines Agency (EMA) has allowed its use as a traditional herbal medicinal product for mild dyspeptic disorders, minor skin inflammation, and for wound healing [4]. Previous studies have presented the constituents of mastic gum essential oil, [5,6] and demonstrated its antimicrobial [6,7,8] and anti-inflammatory properties [9], thus stimulating considerable interest on it as a novel wound healing agent. However, issues like its poor water solubility and volatility, as well as its caustic nature when applied in large quantity, have hindered the development of technological means for cutaneous administration.

In order to overcome these limitations, the rapidly developing field of nanotechnology may be exploited. Effective therapeutic approaches have been developed using nanosystems with cell type specificity, ability of local release of the bioactive substances, and increased safety, due to the sustained release and the selection of biocompatible materials. Investigation of applying nanotechnology for enhancing wound healing process has already been reported [10], proposing effective acceleration of the process through a variety of mechanisms, including the preparation of nanoparticles (NPs) with antimicrobial and anti-inflammatory action [11,12]. Encapsulating essential oils (EOs) into NPs provides a method of protection and controlled release for the oil constituents enclosed in the spherical structures. Although, a variety of EOs has already been encapsulated into NPs [13,14,15,16], there is no report of the inclusion into nanocarriers of EO extracted from the resin of *P. lentiscus* var. *chia*.

In the present study, polymeric NPs have been designed and prepared in order to achieve successful administration of the valuable EO. Poly(lactic acid) (PLA) was chosen as the most appropriate shell material due to its biocompatibility, biodegradability and lipophilic nature, allowing thus the incorporation of oily compounds [17,18]. The surfactant used during NPs preparation is of major importance for the formation and stability of NPs, as it reduces the interfacial tension between aqueous and organic phase, thus allowing formation of the initial emulsion and therefore, of the NPs. It has been shown that the physicochemical characteristics of the surfactant type, such as HLB value, ionic strength and chemical structure play a crucial role with respect to the produced NPs [19,20]. For this reason, different surfactants were tested and the most appropriate of them resulting in nanocarriers with appropriate characteristics was selected.

The physicochemical characteristics of the NPs i.e., particle size distribution, ζ-potential were assessed and the morphology of the NPs has been examined by scanning electron microscopy (SEM). Additionally, the stability and the release rate in vitro has been studied in order to assess their behavior when formulated either in hydrophilic or lipophilic cream for cutaneous application. Finally, the antimicrobial action of net EO and NPs prepared against Gram negative (*E. coli*) and Gram positive (*B. subtilis*) microorganisms has been determined.

## 2. Materials and Methods

### 2.1. Materials

*Pistacia lentiscus* var. *chia* gum essential oil (100% pure, Lot No: 140912) was a kind donation from the Chios Mastic Gum Growers Association (Chios, Greece). Poly (d,l-lactide) Purasorb^®^ PDL 05 (Corbion Purac, Amsterdam, The Netherlands) was a gift from ELTON S.A. (Avlonas, Attica, Greece). Water for injection (WFI) was obtained by Demo S.A., Pharmaceutical Industry (Kryoneri, Attica, Greece). Dichloromethane (DCM), hexane and ethyl acetate (analytical grade) were supplied by Fisher Scientific UK (Leicester, UK). Ethanol (99% denatured with 1% MEK) was obtained from PanReac AppliChem (Darmstadt, Germany). Arlacel^TM^ LC (INCI: sorbitan stearate (and) sorbityl laurate) was purchased from Croda International PC (East Yorkshire, UK). Emulmetik^TM^ 900 (INCI: lecithin) was supplied by Cargill Texturizing Solutions Deutschland GmbH & Co. KG. (Hamburg, Germany), while PEG-40 hydrogenated castor oil was bought from Farmalabor (Canosa de Puglia, Italy). Polyvinyl alcohol (87–90% hydrolyzed, Av. MW 30,000–70,000), Lutrol^®^ F127 (INCI: Poloxamer 407), α-pinene ((–)-α-pinene) purity ≥ 99%, alkane standard solution C_8_–C_20_ (analytical standard, C_8_–C_20_, ~40 mg/L each in hexane), phosphate buffered saline, Mueller Hinton broth and Mueller Hinton agar, resazurin sodium salt and gentamicin sulfate (antibiotic) were purchased from Sigma-Aldrich (Steinheim, Germany). Betadet^®^ HR (INCI: Cocamide propyl betaine) was supplied by Dichem Polymers SA (Acharnes, Attica, Greece). Symsol^®^ PF-3 (INCI: aqua, pentylene glycol, sodium lauryl sulfoacetate, sodium oleoyl sarcosinate, sodium chloride, sodium oleate) was obtained from Symrise AG (Holzminden, Germany). Decane (*n*-decane, 99%) was purchased from Aesar GmbH & Co KG (Benzstrasse, Germany), while aqueous eucerine was purchased from Syndesmos S.A. (Acharnes, Attica, Greece). The microorganisms used were *Escherichia coli* (DSM 1103) and *Bacillus subtilis* sub. *spizizenii* (DSM 347), supplied by The Leibniz Institute DSMZ (Braunschweig, Germany).

### 2.2. Methods

#### 2.2.1. Preparation of EO Loaded NPs

**Figure 1:**
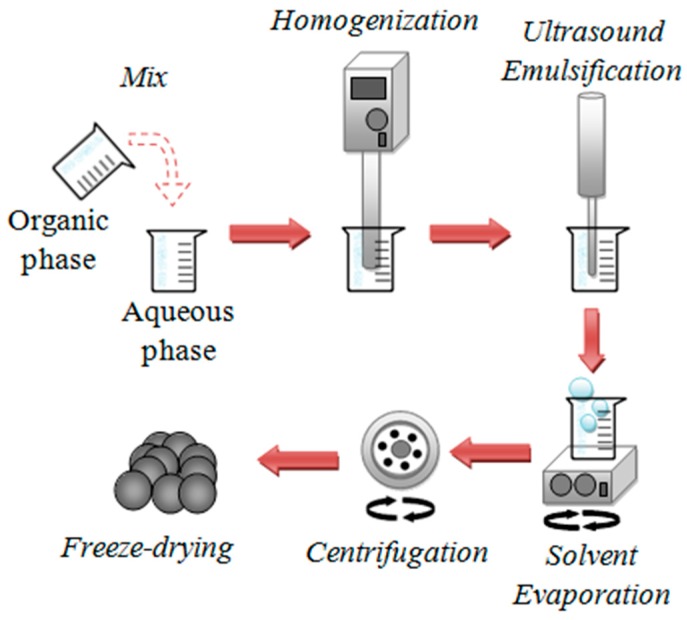
Schematic illustration of NPs-EO preparation.

EO-loaded NPs were prepared utilizing the solvent evaporation/single emulsion technique, as proposed by Vanderhoff et al. in 1979, with slight modifications [21]. Briefly, an aqueous phase was prepared by adding the amount of surfactant (60mg, 0.26% *w*/*w*) in 20 mL of WFI (Figure 1) under stirring (100 rpm, 10 min, RT) on a magnetic stirrer (Magnetic Stirrer Hot Plate, Velp Scientific, Usmate Velate, Italy). The organic phase was prepared separately by mixing 3 mL DCM with PLA (50 mg) and 5 μL EO under vigorous stirring (Vortex-Genie 2, Scientific Industries, Bohemia, NY, USA) for 2 min. When lipophilic surfactants were used (Arlacel ™ LC or Emulmetik^TM^ 900 (LEC) or Betadet^®^ HR) they were added to the organic phase. Thereafter, the organic phase was added to the aqueous one under homogenization (Homogenizer T18D, IKA-Werke, Staufen im Breisgau, Germany) (4000 rpm, 2 min). 

The system was then ultrasonicated (Probe Sonicator Vibracell VCX130 PB, Sonics & Materials, Inc., Newtown, CT, USA) using the following settings: amplitude 40%, 35s, RT, where emulsion formation was observed. The organic solvent was then evaporated by constant mild magnetic stirring under a fume hood. Upon complete removal of the solvent (80 min) the emulsion was converted to a dispersion of solid in aqueous phase, which was collected by centrifugation (Centrifuge Hermle Z32HK, Hermle Labortechnik GmbH, Wehingen, Germany) (13,000 rpm, 15°C, 45 min), followed by rinsing with equal volume of WFI and repeat of centrifugation. The solid was lyophilized (Freeze Dryer FreeZone, Labconco, Kansas City, MO, USA) for 24 h after the addition of sucrose solution (4.0% *w*/*w*) as cryoprotectant, until complete removal of water and stored at 4 °C. For determination of EO encapsulation efficiency (%EE) and loading (%EOL), NPs morphology, in vitro release, and antimicrobial activity assessment, no sucrose was used to allow accurate weighing of dry NPs. All experiments were conducted in triplicate.

#### 2.2.2. Dynamic Light Scattering (DLS) and Electrophoretic Light Scattering (ELS) for Determination of Particle Size, PdI and ζ-Potential

Particle size expressed as hydrodynamic diameter and size distribution expressed as polydispersity index (PdI) were determined using dynamic light scattering (DLS), while ζ-potential measurements were based on electrophoretic light scattering (ELS). All measurements were conducted in a ZetaSizer Nano series Nano-ZS (Malvern Instruments Ltd., Malvern, UK) equipped with a He-Ne Laser beam, set up in a wavelength of 633 nm and backscattering angle of 173°. Settings for ζ-potential measurement contained an average of 100 runs with the phase analysis light scattering mode (PALS) and equilibration temperature of 25 °C. NPs dispersion in water was prepared by dispersing 5 mg of NPs in 10 mL of water. All measurements were carried out in triplicate.

#### 2.2.3. Scanning Electron Microscopy (SEM) for Morphological Characterization of NPs

The morphology of NPs was characterized by scanning electron microscopy (SEM), using an SM 6300 system (JEOL Ltd., Tokyo, Japan) with magnification ability in the range of 10–50000 times and resolution of 3.5 nm (W.D. = 8 mm, 30 kV/SEI). Samples were left to dry under ambient conditions, and subsequently were spatter coated with an Au layer in order to increase electron conductivity.

#### 2.2.4. EO Analysis

Qualitative and quantitative analysis of the EO was performed by Gas Chromatography-Mass Spectrometry (GC-MS) on an Agilent 6890N Gas Chromatographer coupled to an Agilent 5975 B mass spectrometer (Agilent Technologies, Inc., Santa Clara, CA, USA), equipped with an HP-5MS non-polar column (30 m × 0.25 mm × 0.25 μm film thickness). The electron impact ionization source was set at 70 eV and helium was used as carrier gas at flow rate of 1 mL/min. The injected sample volume was 1 μL and the system was set up in splitless injection mode. Injector temperature was set at 300 °C while the source temperature was 250 °C. Initial oven temperature was 60 °C, which increased to 80 °C by a rate of 2 °C/min, where it remained for 2 min. Next, the temperature increased to 110 °C with a rate of 1 °C/min and maintained for 2 min. Finally, with a rate of 15 °C/min, a temperature of 280 °C was reached, which was kept stable for 2 min. The total run time of the method was 57.33 min.

Qualitative analysis was carried out by comparing the obtained MS spectra to literature data [22,23] as well as by retention indices (RI) calculation, based on a series of linear C_8_–C_20_ alkanes using the Van den Dool and Kratz equation. Quantification was accomplished by designing calibration curves using α-pinene in concentration range from 0.005 to 2.0 mg/mL. Decane (0.04 mg/mL) was used as internal standard (IS).

#### 2.2.5. Determination of %EE and %EOL

For the determination of %EE and %EOL of EO in NPs a amount of NPs (18.0 mg for PLA/PVA-NPs, 30.0 mg for PLA/LEC-NPs) was diluted in hexane (600 μL) in a sealed vial under stirring in the dark for 24 h. The supernatant was then harvested and filtered through a filter (0.220 μm, PVDF, EMD Millipore Corporation, Temecula, CA, USA). The NPs were rinsed with 200 μL of hexane under stirring for 10 min and the supernatant was collected in the same manner and added to the previous supernatant. In an 30 μL aliquot of this mixture, 20 μL of IS decane (1.0 mg/mL) were added, resulting in a final concentration of 0.04 mg/mL on a 50 μL total solution GC-MS analysis was conducted as described previously. The %EE and %EOL were determined using Equations (1) and (2), respectively:

(1) $$\%\;EE=\frac{EO\;encapsulated\;in\;NPs}{EO\;added\;during\;preparation\;of\;NPs}\times 100$$

(2) $$\%\;EOL=\frac{EO\;encapsulated\;in\;NPs}{Total\;mase\;of\;NPs\;\left(mg_{PLA}+mg_{EO}\right)}\times 100$$

#### 2.2.6. Stability Study

The prepared NPs were lyophilized and stored either at 25 °C or 4 °C. Their stability was monitored over the period of 60 days, examining particle size, PdI, ζ-potential and %EE at predetermined time intervals.

#### 2.2.7. In Vitro Release Study

The in vitro release rate was evaluated following the dialysis method as proposed by Chidambaram et al. with minor modifications [24]. More specifically, a dissolution method was applied, followed by ultracentrifugation. The required amount of NPs (20.0 mg for PLA/PVA-NPs or 40.0 mg for PLA/LEC-NPs) was dispersed in 5.0 mL of phosphate buffered saline (PBS) solution (0.01 M, pH = 7.4), containing surfactant (Symsol PF-3 3.0% *w*/*w*). Due to the oily nature of the EO, the absence of a surfactant may cause difficulty in entering the aqueous environment. The vial was sealed and placed in a Water Bath Shaker (BioLine Scientific, Athens, Greece) which was set at 100 rpm and 37.0 °C. At predetermined time intervals (1, 3, 6, 24 and 48 h) the dispersion was placed in a refrigerator (4 °C, 10 min) in order to avoid EO loss while unsealing the vial. The supernatant was collected by centrifugation (13,000 rpm, 15 °C, 15 min) and the precipitate was dispersed in 5 mL of fresh PBS-surfactant solution and incubated until next sampling. The EO of the supernatant was extracted in 3 mL of ethyl acetate. Subsequently, 30 μL of the organic solution were obtained and mixed with 20 μL of IS solution (decane in ethyl acetate, 0.1 g/mL) and analyzed by GC-MS. All experiments were performed in triplicate.

Furthermore, the in vitro release study of EO was also conducted in oily environment, by incorporating PLA/PVA-NPs (20.0 mg) in aqueous eucerine (200.0 mg). The mixture was transferred in a dialysis tube (MWCO 12400, Sigma-Aldrich, Steinheim, Germany) and immersed in PBS-surfactant solution. All procedures were conducted as described above, excepting the centrifugation step.

As the procedure contains an extraction step, standard error while calculating the EO quantity was determined, by inserting known amount of EO (5 μL) in the PBS-surfactant solution and analyzing the organic phase after extraction via GC-MS.

In order to quantify the release rate and investigate the release mechanism of the essential oil from the nanoparticles, the release data were fitted to the Korsmeyer-Peppas equation (Equation (3) [25]) using the non-linear regression fitting option of the StatGraphics Plus for Windows, Version 3.3. Manugistics Inc., Rockville, MD, USA:

Q = k·tn(3)

where Q is the fractional release of the drug, k is a constant incorporating structural and geometric characteristics of the drug carrier (dosage form), and n is the release exponent, indicative of the drug-release mechanism.

#### 2.2.8. Evaluation of Antimicrobial Activity

The activity of EO was examined against the growth of *Escherichia coli* (DSM-1103) and *Bacillus subtilis* sub. *spizizenii* (DSM-347) (Gram− and Gram+ species, respectively), using the broth-microdilution method [26]. Antimicrobial activity of EO was determined after dilution in either of two different solvents, ethanol (15.0% *v*/*v* aquatic solution) and DMSO (12.0% *v*/*v*). Concentrations of the organic solvents used were selected after determining the MIC of each one of them, in order to diminish interference with the applied agents.

In particular, eight solutions of diminishing solvent concentration (from 15 to 4 %) were prepared, with the final concentration in each well varying from 7.5 to 2.0%, respectively. Concerning EtOH, no inhibitory effect of the percentages tested was found against the two bacterial species, in accordance with previous findings [27], while in case of DMSO, a final concentration of 7.5% was not clearly non-inhibitory for *E. coli*, and therefore 6.0% was selected for further application.

The final concentration of microorganisms in each well of the microplate (Tissue Culture Testplate 96F, TPP 92696) was 5 × 10^5^ CFU/mL. The method also included a negative control, in which the sample was tested against the presence of microorganisms and a positive control, which monitored the viability of the inoculi used. Furthermore, gentamicin was applied as a reference drug. The microplates were incubated at 35 °C for 15 h. Final interpretation of the results was conducted using resazurin, an indicator of microbial growth, which changes color from blue to pink when reduced in the presence of living cells [28]. Therefore, after 15 h of incubation, 10 μL of resazurin (0.18% *w*/*v*) were added to each well and MIC was determined, after 3 more hours of incubation [29].

#### 2.2.9. Statistical Analysis

Every measurement was performed in triplicates and reported as mean values ± standard deviation (SD). The statistical significance of results was calculated by Student’s t-test using Excel software (Microsoft Office Excel 2007, Redmond, WA, USA).

## 3. Results and Discussion

### 3.1. Preparation of NPs

#### 3.1.1. Optimization of NPs Preparation Conditions

In order to optimize the conditions of NPs preparation, various parameters were altered, resulting in different values for size, PdI and EE content (%EE and %EOL). The surfactant used was PVA and the parameters tested were the surfactant quantity, the sonication amplitude and EO/PLA ratio. The results for each NPs preparation are shown in Table 1.

In order to assess the impact of the quantity of surfactant on the NPs’ parameters, three percentages of surfactant were used, 0.26%, 0.5% and 1.0%. Comparing NPs-1 and NPs-2 preparations a mild reduction in both %EE and %EOL for NPs-2 was observed. This is explained by taking into consideration the increased quantity of surfactant (NPs-2), which resulted in a larger amount of surfactant bound to NPs surface and thus occupying a larger percentage of the total mass of NPs. As a result, when expressing the amount of essential oil present in a particular mass of NPs, this mass was increased while the amount of essential oil remained constant. Further increase of the percentage of surfactant to 1.0% (NPs-3), caused a slight increase in %EE (NPs-3). However, as the excess of surfactant appeared to be unnecessary, the NPs-1 preparation was considered optimal in this comparison. It was also noted that NPs size and PdI did not show any significant differences.

The sonication settings were examined by altering amplitude and time from 40%, 35 s to 60%, 20 s. Among NPs-1 and NPs-4 preparations factors such as size and PdI remained stable, while %EE was remarkably reduced in the second case (38.38% and 28.09%, respectively). This difference is most likely to be attributed to a rise of temperature, caused by increasing sonication amplitude regardless the shorter time period. Hence, a loss of the volatile essential oil could possibly occur, before sonication process was completed and NPs obtain their firmer structure.

The impact of EO content was investigated using three EO/PLA ratios (NPs-5: 1/25, NPs-1: 1/10, NPs-6: 1/7). Compared to NPs-1, preparation of NPs-5 showed an increase in %EE, which however did not agree with %EOL. In particular, %EOL showed smaller amount of EO in the total NPs mass, which appears to be a more realistic indicator than %EE. More specific, %EE calculation has resulted from a division (Equation (1)), which contains the amount of theoretical quantity of EO as denominator, which is reduced, leading to increased results of the division.

Results for NPs-6 preparation showed decrease of %EE (from 38.38% for NPs-1 to 27.98% for NPs-6), while %EOL remained relatively stable, a fact that is again explained through Equation (1), since in this case the denominator was higher, resulting in lower values of the division. These results indicated saturation of NPs with EO and no further loading with essential oil under the specific conditions was possible. It is worth noting that in the preparations altering EO/PLA ratio the PdI factor was increased, although remaining within satisfactory limits (<0.2).

In summary, optimum results were achieved by the preparation of NPs-1. Initially, it was concluded that mean diameter and PdI factors were minimally affected, in comparison with essential oil content. Moreover, the amount of surfactant did not appear to cause any important alteration to the prepared NPs. Furthermore, it was observed that the increase of sonication amplitude resulted in a higher loss of EO, thereby leading to lower %EE and %EOL. Also, the boundaries between the amounts of loading saturation of NPs appeared to be relatively narrow.

#### 3.1.2. Effect of Surfactant Type on NPs Preparation

The influence of the type of surfactant on the properties of the prepared NPs was evaluated, taking into consideration their mean diameter, PdI and ζ-potential as well as the inter-batch repeatability described by the standard deviation. 

Six different non-ionic or amphiphilic surfactants were used, covering a wide range of HLB values (5.5–35). The results of the characteristics for each NP preparation are summarized in Table 2. None of the three factors (size, PdI and z-potential) demonstrated a linear correlation with the HLB value of surfactants. The mean size of all NPs ranged from 239.9 nm to 664.7 nm, while PdIs varied from 0.081 to 0.543. The absolute values of ζ-potential were relatively high indicating satisfactory stability for all surfactants. The smallest mean size was measured for PLA/PVA-NPs (239.9 nm ± 4.3 nm) and PLA/LEC-NPs (286.1 nm ± 6.1 nm) accompanied also by the narrowest distributions as denoted by the PdI (0.081 ± 0.007 and 0.167 ± 0.019, respectively). Similarly, both PVA and LEC, lead to the most satisfactory repeatability for all the three properties. These results lead to the selection of PVA or LEC for the preparation of NPs incorporating EO.

#### 3.1.3. Lyophilization of NPs

The impact of the addition of cryoprotectant (sucrose 4.0% *w*/*w*) as well as lyophilization was studied by measuring the size and PdI of the prepared NPs. The results are summarized in Table 3. Although the addition of the cryoprotectant did not cause significant alterations on the size and PdI of either PLA/PVA-NPs or PLA/LEC-NPs, a decrease in ζ-potential’s absolute value of 52.2% and 15.2% was observed for PLA/PVA-NPs and PLA/LEC-NPs respectively a fact that may indicate destabilization over time in the cases where the absolute value of ζ-potential is less than 20mV. Measuring the size, PdI and ζ-potential after lyophilization, no significant alterations (*p* < 0.05) for PLA/PVA-NPs was found, while the size of PLA/LEC-NPs was increased by 24.2% (*p* < 0.05) a fact that may indicate possible aggregation.

### 3.2. Physicochemical Characterization

#### 3.2.1. Particle Size and ζ-Potential

Both the selected NPs had similar mean particle size (239.9 nm ± 4.3 nm for PLA/PVA-NPs and 286.1 nm ± 6.1 nm for PLA/LEC-NPs), PdI (0.081 nm ± 0.007 nm for PLA/PVA-NPs and 0.167 nm ± 0.019 nm for PLA/LEC-NPs) while absolute value of ζ-potential for both NPs was over 20 (−29.1 mV ± 2.8 mV for PLA/PVA-NPs and −34.5 mV ± 3.7mV for PLA/LEC-NPs) indicating satisfactory stability (Table 2).

#### 3.2.2. Morphology of NPs

**Figure 2:**
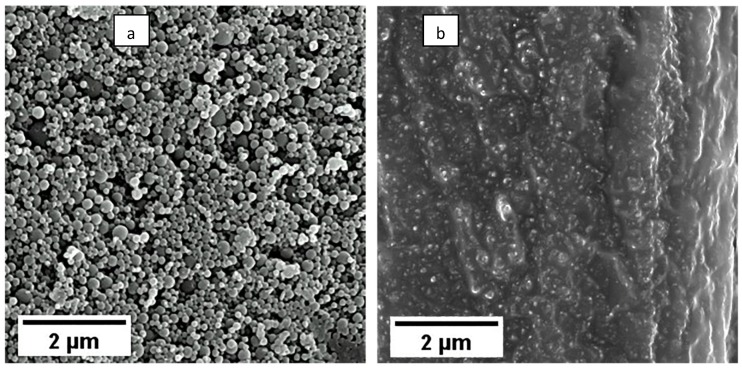
Morphology of (**a**) PLA/PVA-NPs and (**b**) PLA/LEC-NPs determined by SEM.

The morphology of the prepared NPs as evaluated via SEM reveled spherical structures with smooth surface and fine size distribution for PLA/PVA-NPs (Figure 2a), while under the same settings PLA/LEC-NPs revealed some distinctive spherical structures embedded in a fused background (Figure 2b). This morphology is typical of similar lipid-polymer hybrid nanoparticles [30] and may be justified by the fact that due to the presence of LEC excess of water is entrapped in their structure, thus hindering focusing of electron beam on the sample. This fact may suggest existence of different structure between PLA/PVA-NPs and PLA/LEC-NPs and may justify the reduced stability exhibited by the later.

#### 3.2.3. Characterization E.O. Loading in NPs

##### Chemical Composition of EO

The EO ingredients were identified by GC-MS and was found to consist mainly of monoterpenes, while the compounds of higher abundance were α-pinene (82.95%), myrcene (2.85%), β-pinene (2.06%), linalyl acetate (1.29%), *trans*-verbenol (1.36%), linalool (1.00%) and limonene (0.88%) in accordance to literature [5,6]. Due to its high percentage, α-pinene, was used for the quantification of EO in the NPs.

##### Characterization of %EE and %EOL

The %EE and %EOL were determined for PLA/PVA-NPs and PLA/LEC-NPs are summarized in Table 4. The %EE measured for PLA/PVA-NPs was 37.45 ± 1.32, while %EOL was 2.51 (± 0.06). %EE and %EOL for PLA/LEC-NPs was significantly lower (9.15 ± 0.93) and 0.59 ± 0.06, respectively) in comparison to PLA/PVA-NPs. This result indicates that although both NPs had similar particle sizes, combination of PVA with PLA leads to higher loading of EO than combination with LEC.

### 3.3. Stability of EO Loaded NPs

**Figure 3:**
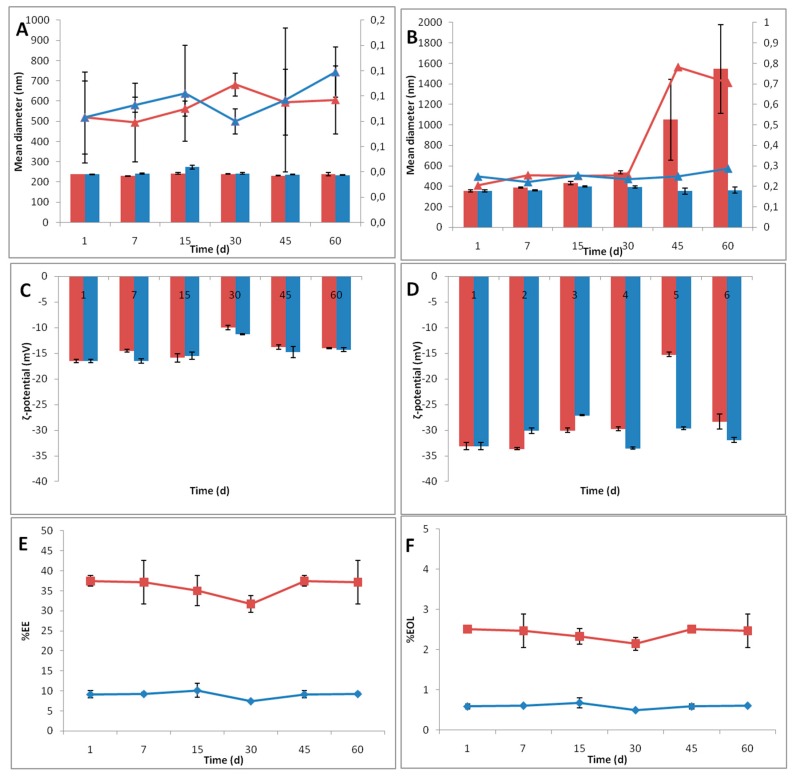
Stability study of PLA/PVA-NPs (**A**,**C**) and PLA/LEC-NPs (**B**,**D**), in storage conditions of 25 °C (red) and 4 °C (blue), monitoring the mean size (■) and PdI (▲) (**A**,**B**), ζ-potential (**C**,**D**). Additionally, %EE (**E**) and %EOL (**F**) of PLA/PVA-NPs (■) and PLA/LEC-NPs (♦) in storage conditions of 4 °C was monitored.

Determining the stability of NPs under different conditions is of great importance, as information regarding products’ behavior at different storage conditions over time can be provided. The results of the stability study of particle size distribution and ζ-potential are presented in Figure 3A–D. PLA/PVA-NPs exhibit structure stability in both temperature conditions tested (25 °C and 4 °C). 

On the contrary, although PLA/LEC-NPs were stable on storage temperature of 4 °C, at 25 °C particles’ mean diameter and PdI increased significantly, most likely due to water sorption by the hydrophilic part of LEC or aggregation of NPs. Despite the fact that ζ-potential was practically unaltered (*p* > 0.05), the dramatic increase (*p* < 0.05) of the particles’, mean size, from 355.4 nm (± 13.2 nm) at day 1, to 1050.0 nm (± 393.1 nm) at day 45 is indicative of reduced stability.

As both PLA/PVA-NPs and PLA/LEC-NPs indicated stable structures in environment of 4 °C, alteration in EO content was studied in this specific temperature. During the period of 60 days, %EE and EOL% did not reduce significantly (*p* > 0.05) for both samples stored at 4 °C (Figure 3E and Figure 3F, respectively).

In summary, although both PLA/PVA-NPs and PLA/LEC-NPs appeared to maintain the content of EO relatively stable, PLA/PVA-NPs seem to have greater prospects for future application due to their stability in storage conditions at both 25 °C and 4 °C.

### 3.4. In Vitro Release of EO

#### 3.4.1. In Vitro Release of EO from NPs Aqueous Dispersion

**Figure 4:**
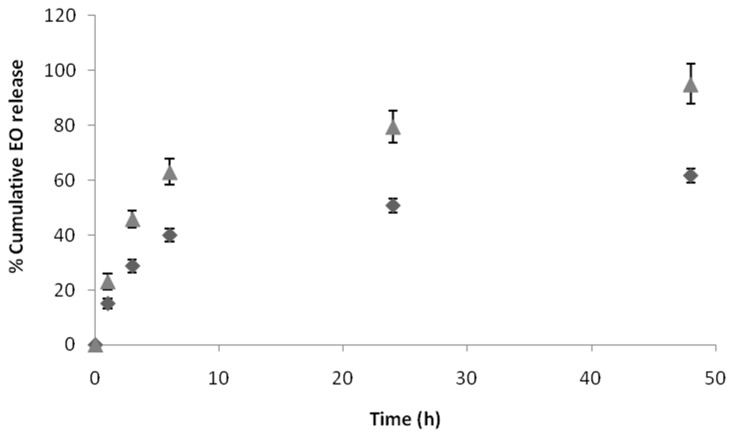
In vitro release of EO from PLA/PVA-NPs (♦) and PLA/LEC-NPs (▲) in PBS (pH = 7.4) at 37.0 °C.

The in vitro release profile of EO from PLA/PVA-NPs and PLA/LEC-NPs was studied under aqueous conditions, as in when, for example, they are formulated as an o/w emulsion or simulating wound environment (pH = 7.4 and 37.0 °C). The percentage of EO released was monitored at predetermined time intervals (1, 3, 6, 24 and 48 h). Two distinct phases were observed for both PLA/PVA-NPs and PLA/LEC-NPs: an initial burst release for the first 6 h followed by more sustained release until the endpoint of the study (Figure 4). This high initial release is desirable for achieving adequate concentration of EO to the point of interest, while a lower release rate could be ideal for further maintaining a sufficient drug concentration at the specific area. Because of the fact that high burst release often is accompanied by a short overall release time, a balance between burst and sustained release is desired in order to achieve optimum pharmacological responses. Ideally a moderate burst release accompanied by a sustained release may provide the desired levels of incorporated substance for a long time period.

Higher burst release was observed for PLA/LEC-NPs, with a percentage of 62.99% released within the first 6 h, while only 39.96% of EO encapsulated in PLA/PVA-NPs was released during the same time period. Furthermore, by the end of the study (48 h) PLA/LEC-NPs had released 95.08% of EO, when at the same time interval the percentage for EO from PLA/PVA-NPs was 61.73%.

#### 3.4.2. In Vitro Release of EO from NPs Dispersed Lipophilic Cream

**Figure 5:**
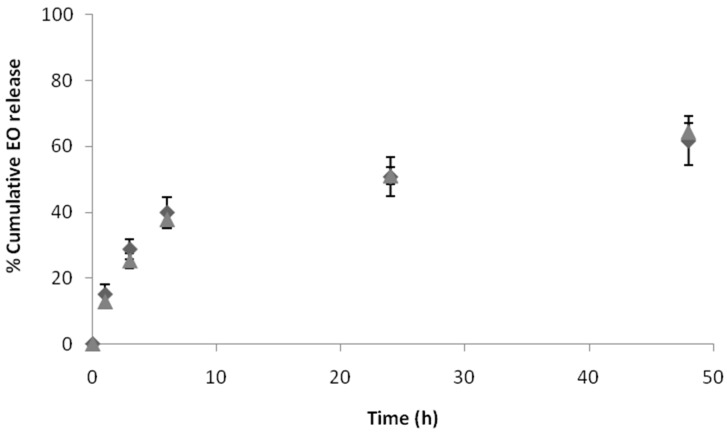
In vitro release of EO from PLA/PVA-NPs in aquatic (♦) or oily environment (▲).

In order to determine whether the environment of NPs affects the release rate of EO when for example they are formulated as an w/o emulsion, a release study was conducted in an oily environment. In particular, PLA/PVA-NPs were introduced into aqueous eucerine a w/o emulsion comprising about 80% oil phase and analyzed for EO released in the same time intervals (Figure 5). It was concluded that PLA/PVA-NPs follow the same release profile, both in aqueous and oily environments. This is an advantage for NPs industrial application, since it does not introduce a limitation for the final formulation, enabling use of different galenic forms of administration (ointment, emulsion, etc.).

#### 3.4.3. Release Kinetics of EO from NPs

Based on the k values obtained by the fitting process (Table 5), the essential oil was released much faster from the PLA/LEC-NPs than from the PLA/PVA-NPs in PBS. This would indicate that PVA formed on the nanoparticles a much less permeable diffusion barrier for essential oil than LEC. The k values obtained also indicate that the essential oil was released at similar rates in the aqueous and the oil release media. The exponent n assumed values between 0.306 and 0.338 (Table 5), suggesting that from both types of NPs and irrespective of the release medium (aqueous or oil) a similar diffusion-dominated release mechanism operated.

### 3.5. Study of Antimicrobial Activity

Minimum Inhibitory Concentration (MIC) was first determined for the pure EO of *Pistacia lentiscus* var. *chia*, in order to subsequently evaluate the activity of NPs, against the growth of *E. coli* and *B. subtilis* (Gram − and Gram +, respectively), under the same experimental conditions. The results of the study are summarized in Table 6. For *E. coli*, MIC in both solvents was found to be 5.00 mg/mL, while for *B. subtilis* a more efficient inhibition of growth was obtained for EO diluted in DMSO, resulting in 1.25 mg/mL, while 2.00 mg/mL were needed in case of ethanol. It is worth noting that the next lower concentration tested for *E. coli* after 5.00 mg/mL, was 2.50 mg/mL, maintaining thus the possibility that actual MIC could be between these two values.

PLA/PVA-NPs were also submitted to the same test in order to determine their potential antimicrobial activity. Because of the turbidity of the final dispersion, the maximum concentration of EO in NPs used in the study was 3.4 mg/mL, as calculated by GC-MS analysis. Therefore, as it was expected from the pure EO’s results, no inhibitory effect on *E. coli* was observed. Similarly, no inhibitory effect was observed in case of *B. subtilis*, although the percentage of EO in NPs exceeded the MIC determined earlier. This observation could be attributed to the sustained release of the EO, since in the first 3 h of the study only a percentage of 30% of the encapsulated EO was released as shown in Figure 4, which corresponds to 1.0 mg/mL. Hence, it is concluded that an extended microbial growth within the first 3 h may occur, which might not be possible to be inhibited by the EO.

*B. subtilis* is a bacterium widely spread in the environment physiologically habitating in the digestive tract, on the skin and therefore it can be found in epithelial wounds. Its adaptive strategies consist of secretion of molecules that may control the growth of other microorganisms such as *Staphylococcus aureus* that may delay the wound healing process. Because of these attributes, probiotic mixtures containing *B. subtilis* are commercially available as skincare products and antibiotic alternative treatment [31]. Based on these results it can be concluded that the incorporation of EO in PLA/PVA-NPs may prevent its antimicrobial action in favor of *B. subtilis* that physiologically inhabits the skin surface or administered topically as an alternative antimicrobial agent [29].

## 4. Conclusions

In this work, PLA-NPs incorporating EO of gum of *P. lentiscus* var. *chia* were prepared. The preparation method was optimized, by applying different experimental conditions, resulting in the design of NPs with improved characteristics in terms of mean diameter, PdI and percentage of encapsulated EO. The effect of surfactant type was evaluated, with PVA and LEC showing optimal results. The morphology study revealed spherical distinctive NPs for PLA/PVA-NPs, while PLA/LEC-NPs NPs were situated in a fussed background. These results, combined with microscopic imaging of NPs, could propose that PLA/PVA-NPs demonstrate structure of nanocapsules, where PLA is the main component of the shell material while EO is enclosed in the core. On the contrary, the results for PLA/LEC-NPs do not indicate a similar structure, due to low EO content, rapid release and a considerable percentage of humidity detected by SEM. These findings suggest the possibility that LEC forms a lipid bilayer which encloses amount of aquatic solution in the core area, while EO is entrapped in the oily shell of the spherical particles. Furthermore PLA/PVA-NPs demonstrated increased stability over time and more sustained release compared to PLA/LEC-NPs. Finally, the antimicrobial activity studies confirm the activity of the essential oil against *E. coli* and *B. subtilis*, while no such results revealed from analogous study conducted in NPs probably due to low concentration of EO at specific time intervals. In conclusion, the results of this research demonstrate a potential use for a novel sustained release system of the *P. lentiscus* var. *chia* essential oil, for cutaneous application when formulated in either hydrophilic or lipophilic cream.

## Figures and Tables

**Table 1:** Optimization of NPs preparation method.

NP Preparations	NPs-1	NPs-2	NPs-3	NPs-4	NPs-5	NPs-6
PLA (mg)	50	50	50	50	50	50
PVA (% *w*/*w*)	0.26	0.5	1.0	0.26	0.26	0.26
EO (μL)	5	5	5	5	2	7
Sonication settings ^a^	40%, 35 s	40%, 35 s	40%, 35 s	60%, 20 s	40%, 35 s	40%, 35 s
Characteristics						
Mean diameter (nm)	239.9	243.2	268.4	256.5	264.9	251.9
PdI	0,081	0.070	0.073	0.081	0.104	0.133
%EE	37.45	36.59	39.36	28.09	51.31	27.98
%EOL	2.51	2.35	2.53	1.90	1.40	2.48

^a^ sonication was conducted in RT.

**Table 2:** Influence of surfactant type on the physicochemical characteristics of NPs.

Surfactant Used for NPs Preparation	HLB	Type of Surfactant	Size (nm)	PdI	ζ-Potential (mV)
Arlacel^TM^ LC	5.5	non-ionic	664.7 (± 313.1)	0.252 (± 0.029)	−31.4 (± 10.8)
Emulmetik^TM^ 900	8	amphiphilic	286.1 (± 6.1)	0.167 (± 0.019)	−34.5 (± 3.7)
PEG-40 hydrogenated castor oil	14	non-ionic	320.5 (± 56.9)	0.314 (± 0.201)	−19.5 (± 7.4)
Polyvinyl Alcohol	18	non-ionic	239.9 (± 4.3)	0.081 (± 0.007)	−29.1 (± 2.8)
Lutrol^®^ F127	22	non-ionic	486.6 (± 197.6)	0.543 (± 0.236)	−20.2 (± 11.5)
Betadet^®^ HR	35	amphiphilic	333.2 (± 138.7)	0.320 (± 0.133)	−34.0 (± 10.7)

**Table 3:** Study of freeze drying conditions on the physicochemical characteristics of NPs.

NP	Addition of Cryoprotectant	Freeze-Drying	Size (± SD) (nm)	PdI (± SD)	ζ-Potential (± SD) (mV)
PLA/PVA-NPs	before	before	239.9 (± 4.3)	0.081 (± 0.007)	−29.1 (± 2.8)
	after		235.3 (± 5.7)	0.089 (± 0.005)	−13.9 (± 0.2)
		after	238.1 (± 0.6)	0.073 (± 0.004)	−16.5 (± 0.3)
PLA/LEC-NPs	before	before	286.2 (± 7.0)	0.163 (± 0.045)	−38.7 (± 0.2)
	after		286.1 (± 6.1)	0.167 (± 0.019)	−32.8 (± 0.5)
		after	355.4(± 13.2)	0.205 (± 0.034)	−33.1 (± 0.7)

**Table 4:** Physicochemical characterization of PLA/PVA-NPs and PLA/LEC-NPs.

Sample	Size (± SD) (nm)	PdI (± SD)	ζ-Potential (± SD) (mV)	%EE	%EOL
PLA/PVA-NPs	238.1 (± 0.6)	0.073 (± 0.036)	−16.5 (± 0.3)	37.45 (± 1.32)	2.51 (± 0.06)
PLA/LEC-NPs	355.4 (± 13.2)	0.205 (± 0.034)	−33.1 (± 0.7)	9.15 (± 0.93)	0.59 (± 0.06)

**Table 5:** Results from fitting the release data to the Korsmeyer-Peppas equation.

Type of NPs	k	n	R^2^
PLA/PVA (in PBS)	19.921	0.308	0.9640
PLA/LEC (in PBS)	31.400	0.306	0.9600
PLA/PVA (in oil medium)	17.636	0.338	0.9673

**Table 6:** MICs determined by broth-microdilution method for EO diluted in organic solvents and incorporated in PLA/PVA-NPs.

Tested Sample	Highest Concentration Tested	Minimum Inhibitory Concentration (mg/mL)	
		*E. coli*	*B. subtilis*
Gentamicin (reference)	2.50 μg/mL	0.25 ± 0.08 μg/mL	0.05 ± 0.02 μg/mL
EO in ethanol 15.0%	5.00 mg/mL	5.00 ± 0.00 mg/mL	2.00 ± 0.00 mg/mL
EO in DMSO 12.0%	5.00 mg/mL	5.00 ± 0.00 mg/mL	1.25 ± 0.00 mg/mL
PLA/PVA-NPs	3.40 mg/mL	> 3.40 mg/mL	> 3.40 mg/mL
